# Multimodal ultrasound tomography for breast imaging: a prospective study of clinical feasibility

**DOI:** 10.1186/s41747-017-0029-y

**Published:** 2017-12-22

**Authors:** S. Forte, S. Dellas, B. Stieltjes, B. Bongartz

**Affiliations:** University of Basel Hospital, Clinic for Radiology and Nuclear Medicine, Petersgraben 4, 4031 Basel, Switzerland

**Keywords:** Breast cancer, Mammography, Multimodal ultrasound tomography (MUT), Three-dimensional (3D), Transmission, Ultrasound

## Abstract

**Background:**

To describe the clinical set-up and evaluate the feasibility of multimodal ultrasound tomography (MUT) for breast imaging.

**Methods:**

Thirty-two consecutive patients referred for breast imaging and 24 healthy volunteers underwent MUT. In the 32 patients, the examination discomfort was compared to that of mammography (n = 31), handheld ultrasound (HUS) (n = 27) and magnetic resonance imaging (MRI) (n = 4) on a scale from 1 (lowest discomfort) to 10 (highest discomfort). MUT investigation time was recorded. Findings automatically detected by MUT were correlated with conventional imaging and biopsy results.

**Results:**

Breast MUT was well tolerated by all 56 participants; 55 bilateral exams were uneventful. During one exam, the digitalisation card failed and the exam was successfully repeated within three days. Mean examination discomfort was 1.6 (range = 1–5) for MUT, 1.5 (range = 1–5) for HUS, 5.3 (range = 3–7) for MRI, and 6.3 (range = 1–10) for mammography. MUT examination time was 38 ± 6 min (mean ± standard deviation). In the patients referred for breast imaging, MUT detected four lesions and indicated malignancy in three of these cases. These findings were confirmed by additional imaging and biopsy.

**Conclusion:**

MUT is feasible in a clinical context considering examination time and patient acceptance. These interesting initial diagnostic findings warrant further studies.

## Key points

- This is the first report on the use of MUT in a clinical setting.
- MUT is feasible in a clinical setting, safe, and well tolerated.
- MUT showed a potential for automated lesion detection and differentiation of breast lesions.

## Background

Breast cancer is the most common malignancy and the leading cause of cancer-related mortality in female population [1, 2]. Since an effective primary prevention of breast cancer is not attainable, the main focus of medical care is on secondary prevention, i.e. early detection. Screening programs based on x-ray mammography are established. However, mammography faces several challenges. First, the density of glandular tissue may obscure lesions; second, it requires a non-negligible radiation dose [3]; and third, breast compression causes patient discomfort. Several studies have demonstrated that for dense breasts, handheld ultrasound (HUS) enables the detection of additional and smaller lesions [2, 4–6], but the time and skill necessary and lack of uniformity has discouraged its widespread use in a screening context. Automated breast ultrasound systems have been proposed as alternative reducing operator dependence compared to HUS [2, 7], but the number of radiologists trained for interpreting this modality is still limited and a comparable recall rate to HUS was reported [7–9].

Thus, a method that can detect breast cancer in an early stage is needed. It should be independent from the glandular density, radiation-free, investigator independent, and cost-effective. In 2011, a novel three-dimensional (3D) imaging technology for detection of breast cancer, multimodal ultrasound tomography (MUT), was introduced [10]. This method uses ultrasound in transmission mode to obtain tomographic images of the breast. Acoustic attributes of each voxel like refractivity, frequency-dependent attenuation, and dispersion can be recorded and combined to yield an automated lesion detection and differentiation [11–14].

However, previous studies reported findings in selected, high-risk women and feasibility has not been evaluated previously. In the present technical development, we describe the clinical setup and evaluate the feasibility of MUT-based breast imaging.

## Methods

This prospective feasibility study was approved by our Institutional Review Board (EKBB196/12) and written informed consent was obtained from all participants. From January till March 2014, we included 24 healthy volunteers (mean age 43 ± 11 years [mean ± standard deviation {SD}], range 27–63 years). Furthermore, within the same time frame, we sought to include patients referred for breast imaging in a consecutive fashion. Exclusion criteria for patients were: mammography or HUS images of the breast older than one month; open wound; breast diameter > 20 cm; lactation; age < 18 years; and lack of informed consent. In three months, 856 patients were referred for breast imaging; 32 (4%) were included (mean age 55 ± 9 years, range 39–76 years). The main reason for exclusion was lack of informed consent. Of these 32 participants, four showed suspicious findings and underwent ultrasound-guided core needle biopsy (14-gauge), one patient had additional magnetic resonance imaging (MRI)-guided (8-gauge) vacuum-assisted biopsy.

**Fig. 1:**
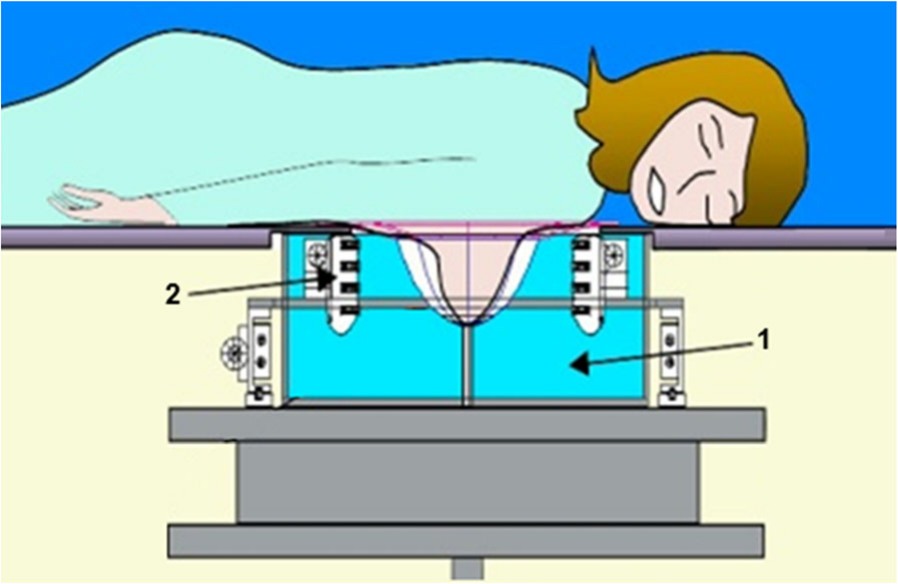
*Schematic image* of patient positioning and setting for MUT. The patient lies prone. The breast is positioned through an opening into the scanning chamber (1). The combined transducer and receiver rotate and scan the breast (2). Courtesy of Mastoscopia

For MUT examination, we used the first available MUT Mark II system (Mastoscopia, Athens, Greece). This improved prototype, compared to a previous version [10, 15, 16] allows a reduction in scan time from 15 min to 9 min per side and increased the field of view from 16 cm to 20 cm. The complete system measures 210 × 130 × 110 cm and weighs 350 kg. The participant was positioned in the prone position and one breast at a time was placed within the scanning chamber which was filled with water. The breast was scanned along the coronal view using ultrasound pulses in transmission mode (Fig. 1).

The MUT imaging system can be divided into two parts: the water conditioner (Onda Corporation, Sunnyvale, CA, USA) and the scanning system. The water conditioner continuously degasifies, de-ionises, heats (kept at 25–30 °C), filters, and ultraviolet (UV)-light irradiates water in the pause between patients to keep the physical attributes of the water constant. This is achieved with a pump (SE-PSH000HM1000, SEKO Spa, Italy) with a flow rate of 2 l/min, which pumps the water from the tank through a 5-μm and 10-μm filter in tandem (Pentek, Milwaukee, WI, USA) as well as a mixed-bed de-ionisation resin filter (Pentek, Sheboygan, WI, USA). For sanitisation purposes, the water passes an UV-C lamp (Puro-Tap, Puro-Parma, Italy). The other cycle transports the water from the tank into the scanning chamber using another pump (SE-PSH000HM1000, SEKO Spa, Italy) with a maximum flow rate of 2 l/min and once more passes an identical UV-C lamp.

The scanning system includes two translating plates (receiver and transmitter) with two columns of 4-mm-sized transducers each (Imasonic, Voray sur l’Ognon, France), which define four coronal scanning planes with an inter-plane distance of 16 mm. The distance between the two columns of transducers is 100 mm. The distance between the plates is 240 mm and transmit–receive transducers are positioned exactly facing each other. The translating motion of plates is driven by two linear motors (PS01 Linmot, Spreitenbach, Switzerland) that control the forward and backward movement. A third motor (Flex Drive Baldor, Fort Smith, AR, USA) rotates the whole scanning chamber continuously from 0 to 180° at each scanning plane and a fourth motor (R88m Omron/Yaskawa, Kioto, Japan) elevates the scanning chamber in steps of 4 mm between scanning planes. This allows 3D scanning in four half-rotations (range of 0–180°) over 64 mm in the vertical/coronal dimension. The opening of the bed is made of flexible Lexan (Sabic, Riyadh, Saudi Arabia) to allow scanning close to the chest wall. This renders the starting scanning position patient-dependent. To standardise this position, the scanning chamber is raised until it touches the Lexan. An automated control system assures the safety of this initialisation, followed by lowering the scanning chamber by 16 mm before starting the scan. A customised electronic pulser device (Mastoscopia, Athens, Greece) generates sequences of broadband ultrasound pulses (frequency range of 1–5 MHz). The first arrival pulse is captured by the receiver and digitised (Octopus GaGe, Lockport, NY, USA).

As these signals are captured at multiple azimuthal-angular positions for each 180° rotation for each coronal plane, an inverse Radon transform allowed the formation of a two-dimensional tomographic image for each acoustic attribute extracted from the received pulse in relation to its transmitted counterpart [11, 12]. The extracted acoustic attributes are the refractivity index, frequency-dependent attenuation, and dispersion. The values of these attributes are normalised by their water-through counterparts in order to obtain a universal validity of these parameters. A specially designed algorithm combines the multimodal information for each pixel to yield a composite image that contains the diagnostically interpretable information. Based on pilot studies [15], the thus derived composite index determines the probability of malignancy within a tissue voxel.

A putative diagnostic threshold of composite index = 1.0 was set for malignant lesions while precancerous conditions corresponded to values in the range of 0.6–1.0, benign lesions to values < 0.6, and normal breast tissue < 0.1 [15]. For improved visualisation, the composite index value was transformed into a diagnostic index (DI) where values from –1.0 to 0.0 represent water (dark blue), values of 0.1–1.5 represent fat (light blue), values of 1.5–3.0 represent normal glandular breast tissue (turquoise/yellow), values of 3.1–5.0 represent benign lesions (orange), and values > 5.0 represent malignancy (red). The different maps were displayed within 3 min after the scan.

Total exam time was measured from entering the door until leaving the examination room.

In the 32 patients, mammography, HUS, and breast MRI were also performed, using standard protocols, meaning for mammography two views of each breast, cranio-caudal and mediolateral oblique views (Selenia Dimensions, Hologic, Bedford, MA, USA), for HUS a linear 15-4 MHz broadband linear array transducer (Aixplorer, SuperSonic Imagine, Aix-en-Provence, France) performed by a radiologist with documentation of at least each quadrant and retromamillar region, and MRI with T2-weighted fast spin echo and T1-weighted dynamic contrast-enhanced images (3 T Skyra, Siemens, Erlangen, Germany).

To these 32 patients, a questionnaire was administered. The exams were rated on a scale of 1–10, where 1 represented no discomfort at all and 10 represented a refusal to repeat. All MUT scans were compared to the imaging available (Table 1). Score distributions of exam discomfort were tested for normality using the Shapiro–Wilk *W* test and significant differences in discomfort between different exam types were evaluated using a two-sided Student’s *t*-test. A significance level of 0.050 was deemed significant. Statistical evaluation was performed using SPSS (Version 22.0). Furthermore, on the experience of the 24 volunteers, all 32 patients were asked to evaluate MUT-specific discomfort on a scale of 0–2 (none, slight and strong), which was pain at the costal arch and neck, but also the temperature of the water.

**Table 1:** Examination discomfort reported by patients for the four modalities

Modality	Mammography	Handheld US	MRI	MUT
Mean score	6.3	1.6	5.3	1.5
Score SD (range)	2.6 (1–10)	1.0 (1–5)	2.1 (3–7)	0.7 (1–5)
Patients	31	27	4	32

*SD* standard deviation

Discomfort level was scored ranging from 1 (no discomfort) to 10 (unwilling to repeat the exam) for mammography, HUS, MRI, and MUT. Indicated are the mean score (second line) and the SD as well as the range of the score (third line). The number of available exams can be found in the last row (patients). Significant differences in discomfort level were found between MUT and mammography (see text)

## Results

Of the 56 participants, 55 were investigated uneventfully with no aborts. One participant had to be re-scanned as a digitisation card failed. Scores for patient discomfort were normally distributed for MUT, HUS, and mammography (*p* = 0.956, 0.660, and 0.693, respectively). MRI could not be evaluated statistically since only four participants underwent MRI. MUT was rated significantly better compared to mammography (*p* = 0.001) and slightly but not significantly better compared to HUS (*p* = 0.205) (Table 1). The time required from entering the room to discussing the results was 38 ± 6 min (mean ± SD). Considering MUT-related patient acceptance, most of the 32 patients referred no or just slight discomfort (Table 2).

**Table 2:** MUT-related discomfort reported by 32 patients

Discomfort	Costal arch	Neck	Temperature
0	26	21	27
1	5	8	4
2	0	1	0
No statement	1	2	1

Exam discomfort was evaluated considering three different aspects (pain at the costal arch, neck and water temperature) ranging from 0 (no discomfort) to 2 (strong). As can be taken from the table, the vast majority experienced no discomfort at all and no participant aborted the scan

**Fig. 2:**
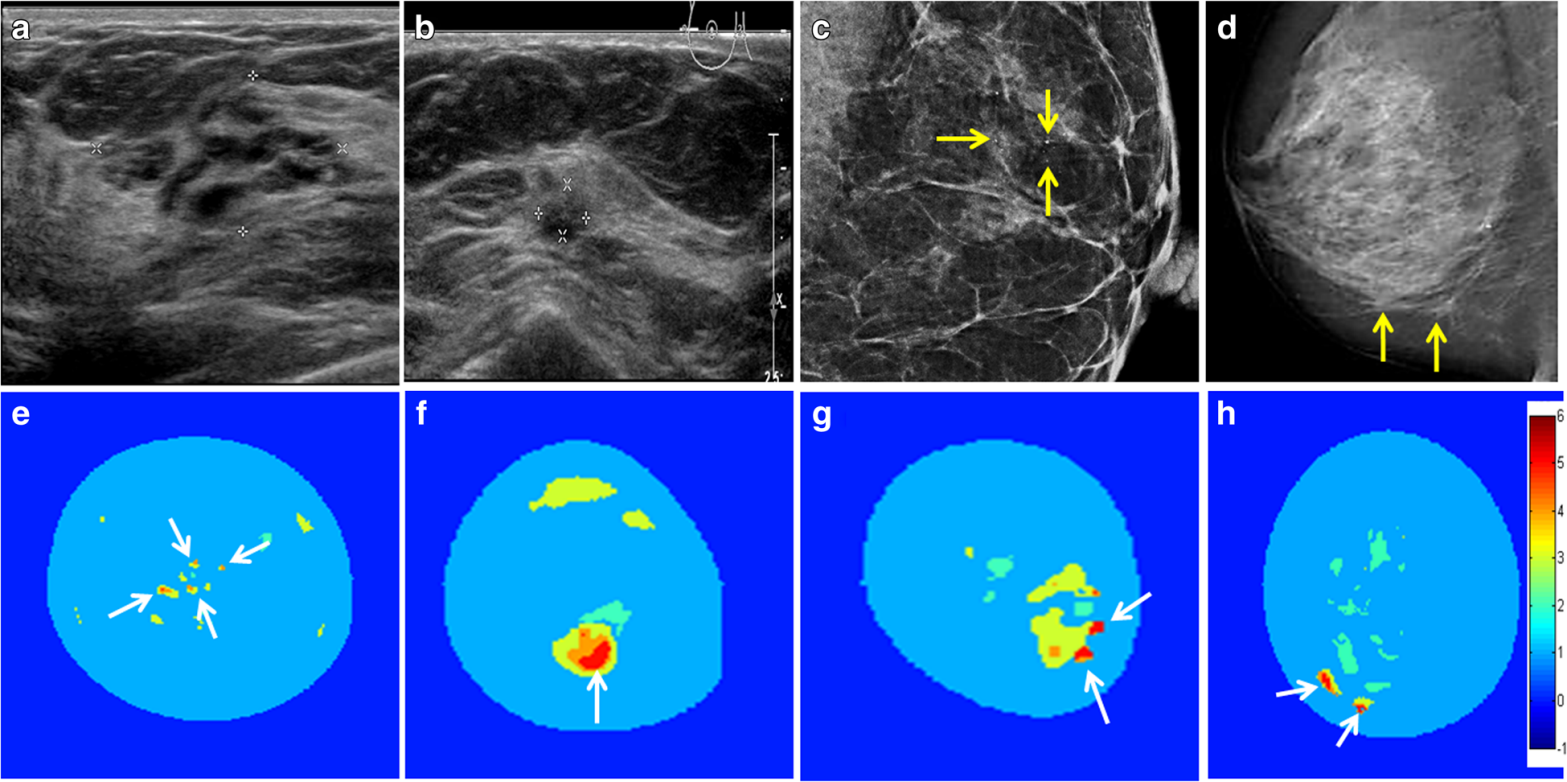
Imaging overview of the four patients with suspicious findings. *Top row* shows HUS (**a**, **b**) and mammography (**c**, **d**); *bottom row* shows the diagnostic index (DI) maps of the MUT exams (**e**–**h**) with the *colour bar* depicting the DI value. **a** A hypoechoic lesion in the right breast, which is lobulated and ill defined. MUT coded this lesion clearly as benign in *orange*, corresponding to a DI value of 4 (**e**, *white arrows*). Histopathology showed a common ductal hyperplasia. **b** HUS of the right breast with an irregular lesion and surrounding distortion of the tissue. MUT coded this lesion as malignant in *red* corresponding to a DI value of > 5 (**f**, *white arrows*). Histopathology demonstrated an invasive ductal carcinoma. Mammography shows in (**c**) regional microcalcifications at 3 o’clock in the left breast (*yellow arrows*). MUT depicted corresponding confined lesions coded *red* (**g**, *white arrows*). Histopathology confirmed an invasive ductal carcinoma with surrounding DCIS. Mammography shows in panel (**d**) two superficial suspicious findings (*yellow arrows*). MUT indicated two findings coded red (**h**, *white arrows*). Histopathology confirmed invasive ductal carcinoma (see also Fig. 3)

**Fig. 3:**
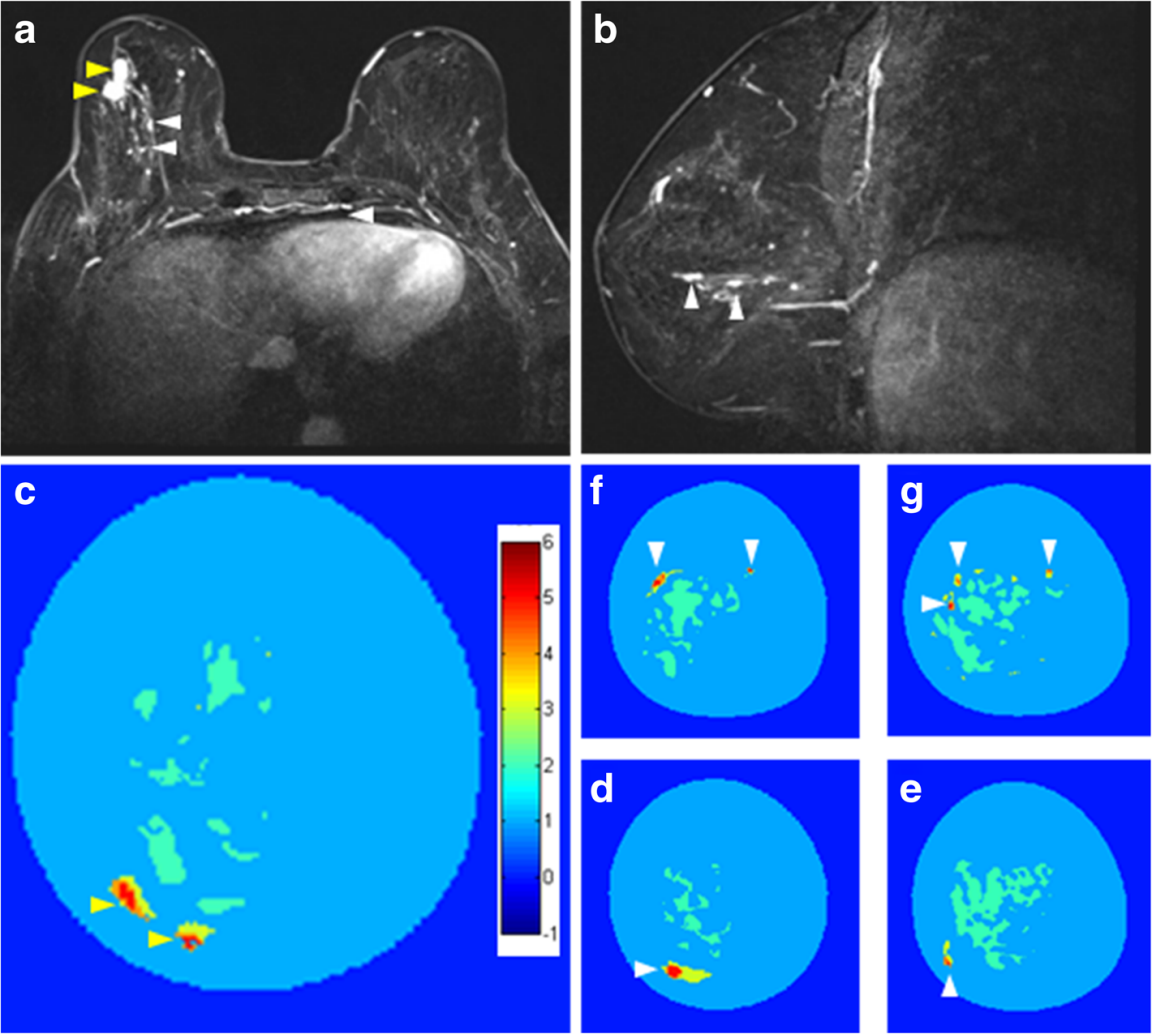
Depiction of a multifocal/mulicentric cancer (same case in Fig. 2d, h). Contrast-enhanced T1-weighted fat-saturated MRI in the axial (**a**) and sagittal reconstructions (**b**). Depiction of multifocal cancer with two masses in the lower outer quadrant (*yellow arrowheads*) and additional ductal enhancement in other quadrants (*white arrowhead*). MUT clearly demonstrates the two masses and coded them correctly in *red* as indicated in **c** (*yellow arrowheads*), but shows also additional small areas with high DI (>5) in the following coronal slices (**d**–**g**, *white arrowheads*). The ductal enhancement was further investigated using an MRI-guided biopsy and the histopathology showed additional DCIS

The reading took just a few seconds as the possible malignancy is marked red. Of the 32 patients, four had a suspicious finding at mammography and/or HUS (Fig. 2a–d) and underwent biopsy. One of the four suspicious findings was proven to be fibrosis with common ductal hyperplasia. The other three findings were proven to be breast cancers: one ductal carcinoma in situ (DCIS) and two invasive ductal carcinomas. As shown in Fig. 2e–h, MUT allowed to detect all four suspicious finding. Furthermore, the benign lesion was correctly coded orange (Fig. 2e) and the three malignancies were correctly coded red (Fig. 2f–h). In one participant, mammography and HUS indicated multiple malignant lesions. This suspicion was fortified using MRI (Fig. 3a, b). Interestingly, initial MUT showed independently both the lesions initially shown by mammography and HUS (Fig. 3c) and additional findings. This linear lesion stretched from the initial finding to the chest wall and a biopsy in this region revealed DCIS (Fig. 3d–g). The other 28 patients had no suspicious findings on mammography and/or HUS as well as on MUT.

## Discussion

Previous pre-clinical studies have shown a potential of MUT for breast lesion detection and characterisation [15–17], but an evaluation of the system in a full clinical setting was lacking. This is the first report using MUT in a clinical setting. Here, we studied up to seven patients per day and there were no major technical difficulties or patient-dependent interruptions of the investigation. The patient setting was well tolerated with just slight discomfort in few patients. Compared to current standard imaging exams, MUT performed comparable or better considering exam comfort. Thus, it seems that in terms of usability, MUT is ready for a day-to-day use in a clinical context. This is clearly the next step and, from our experience, it should be possible to examine approximately eight to ten patients per day. The in-room time is approximately the same required for breast MRI with a full unenhanced and contrast-enhanced protocol. Nevertheless, future optimisation studies may allow reducing it to 30 min. This is also accommodated for by the fact that the size of the system is strongly decreased compared to the initial prototypes. The increased field of view allows the scanning of large breasts and speeding up of the exam increased comfort.

Due to the small number of lesions found in this study, diagnostic performance can only be discussed with the utmost caution. Nonetheless, our results are in accordance with previous reports on the use of the composite index cutoffs for the automated detection of benign and malignant lesions and with the diagnosis made in our routine imaging. Most interestingly, MUT clearly showed in one patient biopsy-proven additional malignant findings otherwise clearly detectable only using MRI (see Fig. 3).

The main focus of this study was to evaluate the feasibility and patient’s acceptance of MUT in a clinical setting. Our patient numbers are too small and our observation times too short to evaluate diagnostic accuracy. In an ongoing study based on this preliminary work, we are aiming to include at least 100 patients per year, including follow-up. This seems feasible since the exam is well tolerated and the set-up is integrated in the breast imaging unit. In this context, we will also need to evaluate the effects on image quality and exam feasibility in a postoperative status. Furthermore, our study was performed in a monocentric fashion and, as such, we cannot estimate the potential challenges for transferring the method to other clinics. The reduced size of the system and automatic detection and classification integrated in the MUT software should aid a seamless integration in other clinical settings; this may soon be evaluated as an additional prototype will become available.

In summary, MUT is feasible in a clinical setting, is safe and well tolerated. The total exam time of about 40 min ensures a substantial patient throughput and first diagnostic findings are encouraging. This warrants larger scale clinical studies both in the context of lesion detection and differentiation.

## Abbreviations

DI: Diagnostic index
HUS: Handheld ultrasound
MRI: Magnetic resonance imaging
MUT: Multimodal ultrasound tomography
SD: Standard deviation

## References

[1] Berry DA, Cronin KA, Plevritis SK (2005). Effect of screening and adjuvant therapy on mortality from breast cancer. New Engl J Med.

[2] Berg WA, Blume JD, Cormack JB (2008). Combined screening with ultrasound and mammography vs mammography alone in women at elevated risk of breast cancer. JAMA.

[3] Svahn TM, Houssami N, Sechopoulos I (2015). Review of radiation dose estimates in digital breast tomosynthesis relative to those in two-view full-field digital mammography. Breast.

[4] Berg WA (2004). Supplemental screening sonography in dense breasts. Radiol Clin North Am.

[5] Crystal P, Strano SD, Shcharynski S (2003). Using sonography to screen women with mammographically dense breasts. AJR Am J Roentgenol.

[6] Kolb TM, Lichy J, Newhouse JH (2002). Comparison of the performance of screening mammography, physical examination, and breast US and evaluation of factors that influence them: an analysis of 27,825 patient evaluations. Radiology.

[7] Chang JM, Moon WK, Cho N (2011). Radiologists' performance in the detection of benign and malignant masses with 3D automated breast ultrasound (ABUS). Eur J Radiol.

[8] Kelly KM, Dean J, Comulada WS (2010). Breast cancer detection using automated whole breast ultrasound and mammography in radiographically dense breasts. Eur Radiol.

[9] Kelly KM, Dean J, Lee SJ (2010). Breast cancer detection: radiologists’ performance using mammography with and without automated whole-breast ultrasound. Eur Radiol.

[10] Zografos E, Koulocheri D, Liakou P et al (2011). Detection of breast cancer via 3D multimodal ultrasound tomography. European Congress of Radiology 2011; poster N. 5349. http://doi.org/10.1594/ecr2011/C-1135

[11] Jeong JW, Kim T-S, Shin DC (2005). Soft tissue differentiation using multiband signatures of high resolution ultrasonic transmission tomography. IEEE Trans Med Imaging.

[12] Jeong JW, Shin DC, Do S (2006). Segmentation methodology for automated classification and differentiation of soft tissues in multiband images of high-resolution ultrasonic transmission tomography. IEEE Trans Med Imaging.

[13] Kim T-S, Do S-H, Marmarelis VZ (2003). Multiband tissue differentiation in ultrasonic transmission tomography, SPIE International Symposium. http://doi.org/10.1117/12.479888

[14] Marmarelis VZ, Kim T-S, Shehada REN (2003). High resolution ultrasonic transmission tomography. SPIE International Symposium. http://doi.org/10.1117/12.479887

[15] Zografos G, Koulocheri D, Liakou P (2013). Novel technology of multimodal ultrasound tomography detects breast lesions. Eur Radiol.

[16] Zografos G, Liakou P, Koulocheri D (2015). Differentiation of BIRADS-4 small breast lesions via multimodal ultrasound tomography. Eur Radiol.

[17] Jeong JW, Shin DC, Do SH (2008). Differentiation of cancerous lesions in excised human breast specimens using multiband attenuation profiles from ultrasonic transmission tomography. J Ultrasound Med.

